# FSH dose to stimulate different patient' ages: when less is more

**DOI:** 10.5935/1518-0557.20170058

**Published:** 2017

**Authors:** Edson Borges Jr., Bianca F Zanetti, Amanda S Setti, Daniela PAF Braga, Rita de Cássia S Figueira, Assumpto Iaconelli Jr.

**Affiliations:** 1 Fertility - Medical Group, São Paulo, SP - Brazil; 2 Instituto Sapientiae – Centro de Estudos e Pesquisa em Reprodução Humana Assistida, São Paulo, SP - Brazil; 3 Disciplina de Urologia, Área de Reprodução Humana, Departamento de Cirurgia, Universidade Federal de São Paulo. - UNIFESP

**Keywords:** FSH, controlled ovarian stimulation, female age, oocyte yield, IVF

## Abstract

**Objective:**

To determine the effect of FSH doses on intracytoplasmic sperm injection (ICSI) outcomes according to the age of the patient.

**Methods:**

Patients undergoing controlled ovarian stimulation (COS) for ICSI cycles in a university-affiliated *in vitro* fertilization center were split into age groups: ≤35 y.o. (n=1523); >35 and ≤38 y.o. (n=652); >38 and ≤40 y.o. (n=332); and >40 y.o. (n=370). The effect of FSH dose on COS, laboratorial and clinical outomes was determined by linear regression models.

**Results:**

The FSH dose didn't affect the ovarian response in terms of total number of follicles, retrieved oocytes and mature oocytes within the age groups, but we found that the lower the age, the lower the FSH dose needed per oocyte retrieved. In the group of patients ≤35 y.o., we also found a positive effect of the FSH dose on oocyte yield. Despite that, for patients ≤38 y.o. there was a negative effect of the FSH dose on embryo quality and blastocyst formation rate, and an increase in the cycle's cancelation rate. In patients ≥39 y.o., there were no effects of the FSH doses on the analysed variables.

**Conclusions:**

Ovarian stimulation with high doses of FSH is not recommended in younger women (≤38 y.o.), once we found a decrease in embryo quality and an increase in cycle's cancelation rate. Mild ovarian stimulation protocols may be more appropriate; however, it may not be applicable for women in advanced age, since a higher FSH dose is needed for oocyte retrieval in these patients.

## INTRODUCTION

*In vitro* fertilization success rates have been remarkably improved since the procedure was first established for clinical use, with the first successful birth in 1978 (Steptoe & Edwards, 1978). Nevertheless, its efficiency, measured as live birth rate, is usually well below 50%. Many couples must undergo several Assisted Reproductive Technology (ART) cycles before they succeed in becoming parents. These multiple trials increase emotional and financial costs, and bring additional risks for women's health (Patrizio & Sakkas, 2009; Seli *et al.*, 2010).

The success of *in vitro* fertilization (IVF) depends on the number and quality of retrieved oocytes and embryos obtained. For that, we employ complex and costly controlled ovarian stimulation (COS) protocols to generate multiple embryos (Fauser *et al.*, 2005). After ovarian stimulation and IVF, the best quality embryos are selected for transfer into the uterine cavity.

Conventional COS regimens routinely use a gonadotrophin-releasing hormone (GnRH) agonist or antagonist to prevent a premature luteinizing hormone (LH) rise, and high doses of exogenous FSH are administered to induce multiple follicle growth (Sunkara *et al.*, 2011; Steward *et al.*, 2014). This traditional COS cause considerable discomfort to the patients, and has important short-term complications, including ovarian hyperstimulation syndrome (OHSS), and a high incidence of multiple pregnancies (Pandian *et al.*, 2009; Setti & Bulletti, 2011). These negative aspects lead to a high rate of drop-outs (Verberg *et al.*, 2008), and increased costs (Goverde *et al.*, 2000). On the other side, a significant proportion of patients show a low or poor response to the classical approach (Bosch & Ezcurra, 2011).

In addition, embryo implantation does not depend exclusively on proper embryo development. It also involves having a receptive endometrium, and a proper synergy with embryos (Dominguez *et al.,* 2003). Growing evidence in the literature shows that COS, with its supraphysiological hormone levels, may decrease endometrial receptivity (Devroey *et al.*, 2004; Shapiro *et al.*, 2011) and embryo quality (Sunkara *et al.*, 2015), increasing embryo implantation failure.

An adequate COS should lead to not only an increased number of retrieved oocytes but also to high quality oocytes and embryos with increased implantation potential, combined with maternal receptive endometrium (Sunkara *et al.*, 2016). However, the response to COS is age dependent and sometimes its unpredictability is a barrier to the implementation of individualized COS protocols (Aanesen *et al.*, 2010; Polyzos *et al.*, 2012).

The determination of the best gonadotrophin dose to be applied for different kinds of patients is extremely important; therefore, the goal of the present study was to establish the effect of different recombinant FSH doses on oocytes and embryo quality, according to patient age.

## METHODS

### Study design

This retrospective cohort study included 12,334 embryos obtained from 2,877 patients undergoing COS, and intracytoplasmic sperm injection (ICSI) cycles. Cycles were performed in a private university-affiliated IVF center, between January/2010 and December/2016. The patients were split into different age groups: ≤35 years old (n=1523); >35 and ≤38 years old (n=652); >38 and ≤40 years old (n=332); and >40 years old (n=370).

The effect of the FSH dose on (i) the number of follicles, (ii) the number of retrieved oocytes; (iii) oocyte yield (number of retrieved oocytes/number of follicles); (iv) number of mature oocytes, (v) mature oocyte rate, (vi) fertilization rate, (vii) embryo quality at cleavage stage, (viii) blastocyst formation rate, (ix) endometrial thickness, (x) cycle's cancelation rate, (xi) implantation rate, (xii) pregnancy rate, and (xii) miscarriage rate, was evaluated on the different age intervals.

The oocytes were evaluated immediately before sperm injection, and the embryos were evaluated 16-18 h post-ICSI and on days three and five of development.

For embryo quality evaluation, the embryos were classified as high or low quality on days three and five.

The implantation rate was defined as the number of gestational sacs divided by the number of embryos transferred per patient. Clinical pregnancy was defined as the presence of a gestational sac with a heartbeat detected by ultrasound 4-6 weeks after embryo transfer.

Written informed consent, in which patients agreed to share the outcomes of their cycles for research purposes, were obtained, and the local institutional review board approved the study.

### Controlled ovarian stimulation

Controlled ovarian stimulation was achieved using a daily dose of recombinant FSH (r-FSH, Gonal-F^®^, Merck KGaA, Darmstadt, Germany) beginning on day three of the cycle. Pituitary blockage was performed using a GnRH antagonist (GnRHa, Cetrotide^®^; Merck KGaA, Darmstadt, Germany), beginning when at least one follicle ≥14 mm was visualized.

Follicular growth was monitored using transvaginal ultrasound, beginning on day four of the gonadotropin administration. When adequate follicular growth, and serum E2 levels were noticed, recombinant hCG (r-hCG, Ovidrel^®^, Merck KGaA, Darmstadt, Germany), was administered to trigger the final follicular maturation. The oocytes were collected 35 hours after hCG administration through transvaginal ultrasound ovum pick-up.

### Preparation of oocytes

Retrieved oocytes were maintained in culture medium (Global^®^ for fertilization, LifeGlobal, Connecticut, USA) supplemented with 10% protein supplement (LGPS, LifeGlobal, Connecticut, USA) and covered with paraffin oil (Paraffin oil P.G., LifeGlobal, Connecticut, USA) for two to three hours before removing the cumulus cells. The surrounding cumulus cells were removed after exposure to a HEPES-buffered medium containing hyaluronidase (80 IU/mL, LifeGlobal, Connecticut, USA). The remaining cumulus cells were mechanically removed by gently pipetting with a hand-drawn Pasteur pipette (Humagen Fertility Diagnostics, Charlottesville, USA).

The oocyte morphology was assessed immediately before sperm injection (4 hours after retrieval) using an inverted Nikon Diaphot microscope (Eclipse TE 300; Nikon^®^, Tokyo, Japan) with a Hoffmann modulation contrast system under 400X magnification. Oocytes that released the first polar body were considered mature and used for ICSI.

### Intracytoplasmic sperm injection

Intracytoplasmic sperm injection was performed in a micro-injection dish prepared with 4-µL droplets of buffered medium (Global^®^ w/HEPES, LifeGlobal, Connecticut, USA) and covered with paraffin oil on the heated stage in an inverted microscope (37.0±0.5°C). Approximately 16 hours after ICSI, fertilization was confirmed by the presence of two pronuclei and the extrusion of the second polar body. Embryos were maintained in a 50-µL drop of culture medium (Global^®^, LifeGlobal, Connecticut, USA), supplemented with 10% protein supplement and covered with paraffin oil in a humidified atmosphere, under 6% CO_2_ at 37ºC for five days.

### Embryo morphology evaluation

Embryo morphology was assessed 16-18 h post-ICSI and on the mornings of days two, three, and five using an inverted Nikon Diaphot microscope (Eclipse TE 300; Nikon, Tokyo, Japan) with a Hoffmann modulation contrast system under 400X magnification.

To evaluate the cleavage-stage morphology, the following parameters were recorded: the number of blastomeres, the percentage of fragmentation, the variation in blastomere symmetry, the presence of multinucleation and the defects in the zona pellucida and cytoplasm. The high-quality cleavage-stage embryos were defined as those with the following characteristics: 4 cells on day two, or 8−10 cells on day three, <15% fragmentation, symmetric blastomeres, the absence of multinucleation, colorless cytoplasm with moderate granulation and no inclusions, the absence of perivitelline space granularity and the absence of zona pellucida dysmorphism. Embryos lacking any of these characteristics were of low quality.

The size and compactness of the ICM and the cohesiveness and number of TE cells were recorded to evaluate the blastocyst-stage morphology. The embryos received a numerical score from one to six, based on their degree of expansion and hatching status as follows: 1, an early blastocyst with a blastocele that was less than half of the volume of the embryo; 2, a blastocyst with a blastocele that was greater than half of the embryo volume; 3, a full blastocyst with a blastocele that filled the embryo; 4, an expanded blastocyst; 5, a hatching blastocyst; and 6, a hatched blastocyst. The ICM of full, expanded, hatching, and hatched blastocysts were classified as either high quality (tightly packed with many cells) or low quality (loosely grouped with several or few cells). Similarly, TEs were classified as either being of high quality (many cells forming a cohesive epithelium) or low quality (few cells forming a loose epithelium or very few cells).

Embryo transfer was performed on the fifth day of development.

### Clinical Follow-up

A pregnancy test was performed 12 days after embryo transfer. All women with a positive test were submitted to a transvaginal ultrasound scan 2 weeks after the positive test. Clinical pregnancy was diagnosed when the fetal heartbeat was detected. Pregnancy rates were calculated per transfer. Miscarriage was defined as pregnancy loss before 20 weeks.

### Statistical analyses

The FSH dose effect on the number of follicles, number of retrieved oocytes, oocyte yield, number of mature oocytes, mature oocyte rate, fertilization rate, embryo quality at cleavage stage, blastocyst formation rate, endometrial thickness, cycle's cancelation rate, implantation rate, pregnancy rate, and miscarriage rate at different age intervals were evaluated by linear regression on the different age intervals (≤35 years old, >35 and ≤38 years old, >38 and ≤40 years old and >40 years old). The results are expressed as regression coefficients (RC) and *p* value.

The FSH/oocyte ratio was calculed as FSH total dose (UI) per number of retrived oocytes. The effects of the age groups on this variable were determined by One-way ANOVA. The results are expressed as mean and *p* value.

All the statistics analyses were performed at the IBM SPSS 20 Software.

## RESULTS

A total of 12,334 embryos obtained from 2877 patients undergoing the first ICSI cycles were included in the analysis. The descriptive analysis of the patients' demographics and COS outcomes is shown in Table 1. Laboratorial and clinical outcomes are described in Table 2.

**Table 1:** Descriptive analysis of patient demographics and COS outcomes.

Variable	General		≤35 years old		36-38 years old		39-40 years old		>40 years old	
	Mean	SD	Mean	SD	Mean	SD	Mean	SD	Mean	SD
**Patient’s demographics**										
Maternal age (y)	35.1	4.5	31.7	2.8	36.9	0.8	39.4	0.5	42.6	1.8
Paternal age (y)	38.0	6.6	35.7	5.9	39.3	6.1	41.3	6.0	43.0	6.7
Total FSH administred (IU)	2318.8	607.9	2185.9	580.2	2442.7	574.5	2508.9	621.0	2476.9	637.4
**COS outcomes**										
Aspirated follicles (n)	16.5	12.1	15.0	9.8	14.6	10.7	11.0	8.0	8.0	6.8
Retrieved oocytes (n)	11.8	9.2	14.9	19.8	10.2	8.0	7.7	6.2	5.6	5.3
Oocyte Yield (%)	70.3	22.4	72.5	19.8	68.5	22.7	66.9	25.1	67.5	28.2
Mature oocytes MII (n)	8.9	7.0	11.2	7.5	7.7	6.0	6.0	4.8	4.3	4.0
Mature oocytes rate (%)	74.5	21.0	75.1	18.5	71.4	22.4	74.5	22.8	72.8	26.6

Note: COS: controlled ovarian stimulation; FSH: follicle-stimulated hormone; SD: standard deviation.

**Table 2:** Descriptive analysis of laboratorial and clinical outcomes.

Variable	General		≤ 35 years old		36-38 years old		39-40 years old		>40 years old	
	Mean	SD	Mean	SD	Mean	SD	Mean	SD	Mean	SD
**Laboratory outcomes**										
Fertilization rate (%)	83.3	19.5	86.1	17.3	83.2	19.9	81.9	23.4	82.1	23.9
High quality D3 embryos (n)	3.1	2.9	3.8	3.2	2.8	2.8	2.2	2.3	1.6	1.7
High quality Embryo rate (%)	45.8	31.1	48.1	28.6	42.7	32.4	42.0	32.8	45.4	37.1
Blastocyst formation rate (%)	41.1	28.4	44.3	27.9	36.6	28.1	41.3	29.7	27.5	26.9
Transferred embryos (n)	1.5	0.6	1.6	0.6	1.5	0.7	1.5	0.7	1.4	0.7
Endometrium (mm)	11.0	7.0	11.1	5.7	10.9	6.6	10.9	2.2	11.3	13.6
**Clinical outcomes **										
Cancelation rate (%)	22.4		20.4		20.7		24.7		32.7	
Implantation rate (%)	29.5		34.9		26.7		28.9		10.7	
Clinical pegnancy rate (%)	38.7		45.6		35.1		24.7		14.0	
Miscarriage rate (%)	13.6		11.0		16.5		17.9		28.6	

Note: SD: standard deviation.

The FSH dose effect was evaluated though linear regression models in different age groups (Tables 3, 4 and 5). The FSH dose didn't affect the ovarian response to COS (i.e. aspirated follicles, retrieved oocytes, and mature oocytes); however, a positive effect on oocyte yield (number of retrieved oocytes/number of follicles) in the patients ≤35 years old could be seen. Despite that, we found a negative effect of FSH on cleavage stage embryo quality and blastocyst formation rate, for patients ≤38 years old (both groups: ≤35 and 36-38 years old). In these groups of patients, the cycle's cancelation rate was positivelly affected by the FSH dose. When analysing the cause of cancellation, we found that the main cause was OHSS risk, 89% and 82.7%, for patients ≤35 an 36-38 years old, respectively.

**Table 3:** Linear regression models of the effect of FSH dose on COS outcomes, at different maternal age intervals.

Group	≤35 years old		36-38 years old		39-40 years old		>40 years old	
Variable	RC	*p*	RC	*p*	RC	*p*	RC	*p*
**COS outcomes**								
Aspirated follicles	-0.004	NS	-0.003	NS.	0.000	NS	0.001	NS
Retrieved oocytes	-0.002	NS	-0.001	NS	0.000	NS	0.000	NS
Oocyte Yield	0.003	**0.002**	0.003	NS	0.003	NS	0.000	NS
Mature oocytes MII	-0.002	NS	-0.001	NS	0.000	NS	0.000	NS
Mature oocytes rate	-0.001	NS	-0.002	NS	0.002	NS	-0.001	NS

Note: COS: controlled ovarian stimulation; RC: regression coefficient, NS: non-significant (*p*>0.05).

**Table 4:** Linear regression models of the effect of FSH dose on laboratorial outcomes, at different maternal age intervals.

Group	≤35 years old		36-38 years old		39-40 years old		>40 years old	
Variable	RC	*p*	RC	*p*	RC	*p*	RC	*p*
**Laboratory outcomes**								
Fertilization rate	-0.001	NS	0.000	NS	0.001	NS	-0.002	NS
High quality D3 embryos	-0.001	NS	0.000	NS	0.000	NS	0.000	NS
High quality Embryo rate	-0.005	**<0.01**	-0.006	**0.025**	0.002	NS	-0.002	NS
Blastocyst formation rate	-0.005	**<0.01**	-0.007	**0.042**	0.004	NS	-0.004	NS
Transferred embryos	0.000	**<0.01**	0.000	NS	0.000	NS	0.000	NS
Endometrium	0.000	NS	0.000	NS	0.001	NS	0.000	NS

Note: RC: regression coefficient, NS: non-significant (*p*>0.05).

**Table 5:** Linear regression models of the effect of clinical outcomes, at different maternal age intervals.

Group	≤35 years old		36-38 years old		39-40 years old		>40 years old	
Variable	RC	*p*	RC	*p*	RC	*p*	RC	*p*
**Clinical outcomes **								
Cancelation rate	0.000	**0.022**	0.000	**0.039**	0.000	NS	0.000	NS
Implantation rate	-0.002	NS	-0.004	NS	-0.001	NS	0.001	NS
Clinical pegnancy rate	0.000	NS	0.000	NS	0.000	NS	0.000	NS
Miscarriage rate	0.001	NS	0.002	NS	0.001	NS	0.003	NS

Note: RC: regression coefficient, NS: non-significant (*p*>0.05).

In patients ≥39 years old, there were no effects of the FSH doses on the analysed variables.

**Figure 1:**
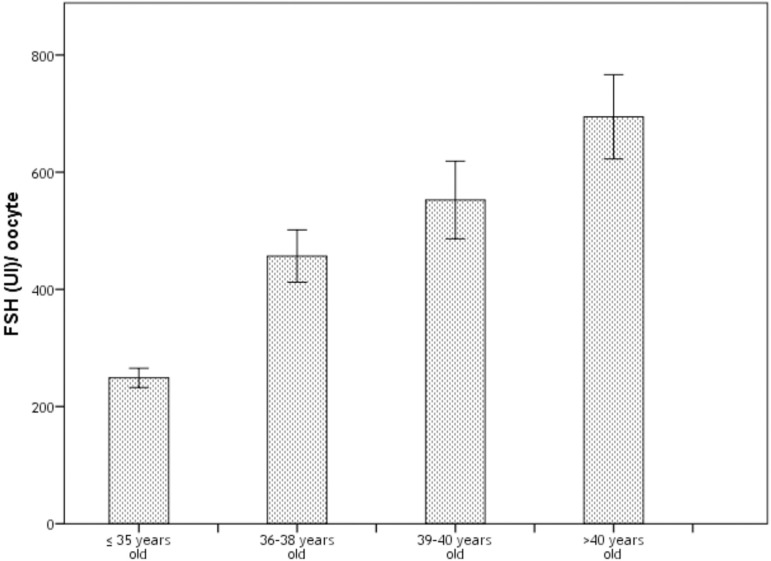
FSH per oocyte ratio. The mean FSH needed for one oocyte retrievel is represented in the bars. The error bars represent 95% CI.

The FSH/oocyte ratio (calculed as FSH total dose (UI)/ number of retrived oocytes) was significantly higher over the years (Table 6), which means that a higher FSH dose is need for oocyte retrieval in older women (Figure 1).

**Table 6:** One-way ANOVA of the effect of age groups on FSH per oocyte ratio.

Group	≤35 years old		36-38 years old		39-40 years old		>40 years old		
Variable	Mean	SD	Mean	SD	Mean	SD	Mean	SD	*p*
FSH/oocyte ratio	248.99	334.72	456.59	590.53	552.59	662.94	694.59	725.88	<0.05

Note: FSH: follicle-stimulated hormone; SD: standard deviation.

## DISCUSSION

An optimal response to COS is crucial for the success of IVF. Either too low or too high ovarian responses to gonadotrophin stimulation are associated with increased cycle's cancellation rates and lower pregnancy rates. Previous reports have suggested an optimal range of oocytes below and above which outcomes are compromised (van der Gaast *et al.*, 2006; Sunkara *et al.*, 2011). However, the ideal dose of FSH for COS which results in optimal oocyte recovery and proper embryo development is still uncertain. This depends on many variables, but the patient's age seems to be the principal factor that affects ovary response to stimulation.

For the present study, the FSH dose effects on laboratorial and clinical ICSI outcomes were evaluated through linear regression models in different age groups. Intriguingly, no significant effect of FSH dose on the ovary response to COS (i.e. aspirated follicles, retrieved oocytes and mature oocytes), could be noted. However, for younger patients (≤35 years old) we found a direct correlation between the FSH dose and the oocyte yield (oocyte retrieval rate).

Concerning the embryo development, for patients ≤38 years old (both groups: ≤35 and 36-38 years old), the increased FSH dose had a negative impact on both cleavage stage embryo quality rate and blastocyst formation rate. Moreover an effect on cycles cancelation risk was noted, in which the higher the dose of FSH, the greater the chance of cancellation.

During COS, the supraphysiological doses of gonadotrophins maintain FSH and LH levels above a critical threshold needed to stimulate the development of multiple follicles, enabling the retrieval of many oocytes in a single IVF cycle (Fatemi *et al.*, 2012).

Conventional ovarian stimulation protocols used to maximize the number of collected oocytes and therefore yielding more embryos, has been associated with good clinical outcomes, in the last decades. However, high doses of gonadotrophins may lead to increased patient discomfort and higher costs (Bodri *et al.*, 2008; Verberg *et al.*, 2008; Steward *et al.*, 2014). Moreover, concerns on the embryo developmental potential have been raised.

Although previous reports have demonstrated that higher doses of gonadotrophins used in conventional stimulation protocols are not likely to have a negative effect on embryo quality (Kok *et al.*, 2006) or embryo aneuploidy incidence (Ata *et al.*, 2012; Labarta *et al.*, 2012), other have demonstrated the opposite (Ziebe *et al.*, 2007; Haaf *et al.,* 2009; Braga *et al.*, 2012), which corroborates with our findings. In the present study, no significant effect on pregnancy was observed, but other studies suggested that the increase in gonadotrophins is also associated with lower pregnancy rates (Jenkins *et al.*, 1991; Stadtmauer *et al.*, 1994; Shulman *et al.*, 1995) .

In recent years, mild stimulation protocols have risen in popularity. Typically, these protocols use lower doses of gonadotrophins and/or shorter stimulation protocols, aiming to retrieve less than eight oocytes (Fauser *et al.* 2010). Even though some studies have shown that the pregnancy rate per cycle is lower, milder stimulation may result in the retrieval of good quality oocytes, which, thereafter, may result in high quality embryos with increased implantation potential (Hunault *et al.*, 2002; Hohmann *et al.*, 2003; Baart *et al.*, 2007).

In our study, an increased rate of cycle's cancelation was correlated with higher FSH doses in younger patients. Our evidence also showed that, especially in this group of women, the risk of OHSS is even higher. A gold standard COS should result in a sufficient number of retrieved oocytes, deviating from both undesirable results, OHSS and poor response. The question that rises is: "Should mild stimulation be applied for everyone?"

This could be of concern to older patients. Although the cumulative pregnancy rate (from fresh and frozen transfers from a single cycle, or from cumulative IVF cycles) was shown to be comparable with both approaches (Heijnen *et al.*, 2007; Fatemi *et al.*, 2013), this may not be a reality for everyone. Indeed, the development of less costly and more comfortable and practical protocols for COS is of pivotal importance, however, in developing countries, in which IVF treatments do not qualify for reimbursements, these protocols may not be a suitable alternative, and sometimes a single treatment may be the only option for these patients.

Our results suggested that in older patients, embryo quality and the blastocyst formation rates was not compromised by increased FSH doses. It could be argued that with advanced age, in general, the embryo quality is already impaired, and probably if there was a negative effect of FSH in the embryo biology, this was disguised in this group. On the other hand, to start the COS with lower FSH doses in older patients may not be an alternative, since it may lead to an increased rate of embryo transfer cancellation due to an insufficient number of oocytes retrieved.

As demonstrated here, the efficiency, in terms of FSH dose per retrieved oocyte, is much better in young patients. Then, mild stimulation protocols may be an alternative for younger women, since although higher FSH doses may result in increased oocyte yield, it significantly impacts the embryo developmental potential.

Lower FSH doses seem to be a great alternative to decrease discomfort, costs, and stress. Moreover, a mild ovarian response may be more appropriate for oocyte biology and it also works better in modern IVF protocols. With this approach, decreased number of embryos transferred and undesirable multiple pregnancies may be achieved, in addition, it results in less surplus embryos, and the inconvenience that it may cause, for storage. However, it may not be applicable for all patients, especially patients in advanced age.

In conclusion, mild ovarian stimulation protocols may be more appropriate for younger women (≤38 years old), since our results indicated that, in these patients, there is a decrease in embryo quality and an increase in cycle's cancelation rate with the increase of FSH dose. However, this approach may not be useful for older woman (≥38 years old), given that higher FSH doses are needed to induce an adequate ovarian response, and this increase was not associated with impaired embryo development.
